# *Plasmodium falciparum* gametogenesis essential protein 1 (GEP1) is a transmission‐blocking target

**DOI:** 10.1002/1873-3468.70184

**Published:** 2025-10-13

**Authors:** Frederik Huppertz, Milagros Siebeck Caturelli, Lina S. Lehmann, Florian Kurth, Alexander G. Maier, Kai Matuschewski

**Affiliations:** ^1^ Department of Molecular Parasitology, Institute of Biology Humboldt University Berlin Germany; ^2^ Research School of Biology The Australian National University Canberra Australia; ^3^ Department of Infectious Diseases and Critical Care Medicine Charité ‐ Universitätsmedizin Berlin Germany; ^4^ Centre de Recherches Médicales de Lambaréné Gabon

**Keywords:** exflagellation, gametogenesis, malaria, *Plasmodium*, transmission, transport protein

## Abstract

Transmission of *Plasmodium* parasites to *Anopheles* mosquitoes relies on rapid activation of mature gametocytes in the midgut, triggered by a temperature drop and xanthurenic acid. In *Plasmodium yoelii*, the gametogenesis essential protein 1 (GEP1) was linked to xanthurenic acid (XA)‐dependent gamete activation. We characterized *GEP1* in *Plasmodium falciparum* using CRISPR‐Cas9 to create *PfGEP1* loss‐of‐function lines. These lines failed to undergo male or female gametogenesis, even when stimulated by XA or a temperature drop. The defect persisted despite treatment with the phosphodiesterase inhibitor Zaprinast. Analysis of field samples revealed two GEP1 single‐nucleotide polymorphisms (V241L and S263P) in 12% and 20% of 49 cases. Our findings confirm GEP1's essential role in gamete activation, highlight an XA‐independent function, and support its potential as a transmission‐blocking target.

**Impact statement**

For sustainable malaria control, transmission‐blocking drug targets are urgently needed. Work in murine models showed that GEP1 is a candidate. We show complete block of life cycle progression of the human malarial parasite *Plasmodium falciparum* when GEP1 is deleted, warranting targeted drug development to achieve gamete‐free mosquito blood meals.

## Graphical abstract

This study shows *Plasmodium falciparum* GEP1 is vital for activating sexual stages of malarial parasites even independently of a mosquito factor. Knockout parasites completely fail gamete formation even when a phosphodiesterase inhibitor is added. Two single‐nucleotide polymorphisms (V241L and S263P) are found in 12%–20% of field samples. GEP1 is a strong transmission‐blocking drug target.

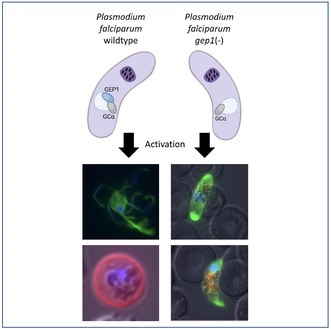

## Abbreviations

**CDPK**, calcium‐dependent protein kinase

**cGMP**, guanosine 3′,5′‐cyclic monophosphate

**GABA**, gamma‐aminobutyric acid

**GCα**, guanylyl cyclase alpha

**GEP1**, gametogenesis essential protein 1

**ICM1**, protein important for calcium mobilization 1

**PDE**, phosphodiesterase

**PI‐PLC**, phosphoinositide‐specific phospholipase C

**SNP**, single‐nucleotide polymorphism

**WT**, wild‐type

**XA**, xanthurenic acid

Malaria remains the most important arthropod‐borne infectious disease with an estimated 263 million infections and 597 000 deaths per year [1]. *Plasmodium* parasites, the causative agent of malaria, follow a complex developmental program in the vertebrate host, where they can cause life‐threatening disease, and the *Anopheles* vector, where sexual recombination takes place. Accordingly, maturation of the sexual precursor cells in the human blood, termed gametocytes, represents a potential point of attack for transmission intervention strategies, which are considered pivotal for malaria elimination [2].

Blood‐stage parasites repeatedly infect and replicate inside erythrocytes and eventually commit to sexual stages [3]. Maturation of *P. falciparum* gametocytes typically occurs over the course of 10–12 days and is commonly divided into five morphologically distinct stages (I–V) [4, 5]. Whereas the early Stage I gametocytes are almost indistinguishable from their asexual counterparts, starting from Stage II gametocytes increase in volume and begin accumulating hemozoin pigment in a sex‐dependent manner. Female gametocytes exhibit condensed hemozoin crystals while male parasites distribute the crystals more scattered throughout the cell. These differences become more apparent as gametocytes develop further. During Stages III to V, parasites elongate and give the host cell the characteristic sickle‐cell, so‐called falciform, shape that the species derives its name from. Ultimately, gametocytes serve the purpose of transmission from the human host back into the mosquito vector.

Once gametocytes are taken up by a mosquito during its blood meal, they are activated by the drop in temperature and a rise in pH, as well as the presence of xanthurenic acid (XA) in the mosquito midgut [6, 7]. How exactly these stimuli translate into intracellular activity is not yet fully understood, but shortly after transmission an intracellular signaling cascade commences, beginning with an increase in guanosine 3′,5′‐cyclic monophosphate (cGMP) concentration mediated by guanylyl cyclase alpha (GCα) [8, 9]. cGMP is subsequently used by a protein kinase (PKG) to phosphorylate a multipass membrane protein, termed important for calcium mobilization‐1 (ICM1), which was suggested as a Ca^2+^ channel that mediates Ca^2+^ release from internal storages [10]. Phosphoinositide‐specific phospholipase C (PI‐PLC) is known to be required for this Ca^2+^ mobilization [11], but the interactions between PI‐PLC, ICM1, and Ca^2+^ remain unresolved. Downstream of the Ca^2+^ release, several calcium‐dependent protein kinases (CDPKs) and calcineurin regulate egress events. There is some overlap between male and female gametogenesis, including the reliance on CDPK1 to mediate egress from the host cell [12]. In contrast, CDPK2 appears to function only in male gametocyte egress [13]. Finally, eight microgametes are released by the male gametocyte that can fertilize a macrogamete formed by the egressed female gametocyte.

Recently, a candidate transport protein, termed gametogenesis essential protein 1 (GEP1), was identified in the rodent malaria model parasite *Plasmodium yoelii* as an essential component of gametocyte activation [14]. GEP1 colocalizes with GCα and was suggested to be needed for its activity. Parasites deficient in *PyGEP1* did not exhibit increased cGMP concentrations, which is typically detected upon stimulation with xanthurenic acid, indicating that GEP1 may function upstream of cGMP in the signaling cascade [14]. This defect could not be bypassed with the phosphodiesterase (PDE) inhibitor Zaprinast, which has been shown to trigger gametogenesis in wild‐type (WT) parasites [15]. Upon Zaprinast inhibition the basal activity of GCα eventually increases cGMP levels above the threshold without further activation, since the counteracting PDE activity is inhibited. The lack of Zaprinast‐induced gametogenesis in *PyGEP1*‐deficient parasites supports the notion that GEP1 is needed for this basal activity of GCα. While the exact role of GEP1 in gametogenesis remains unknown, the complete defect of *gep1(−)* parasites in gamete egress entitle GEP1 as an attractive target to prevent parasite maturation in the mosquito vector. Whether *GEP1* defects reproduce in *P. falciparum* and, hence, whether it qualifies as a candidate drug target awaits experimental genetics confirmation.

**Fig. 1:**
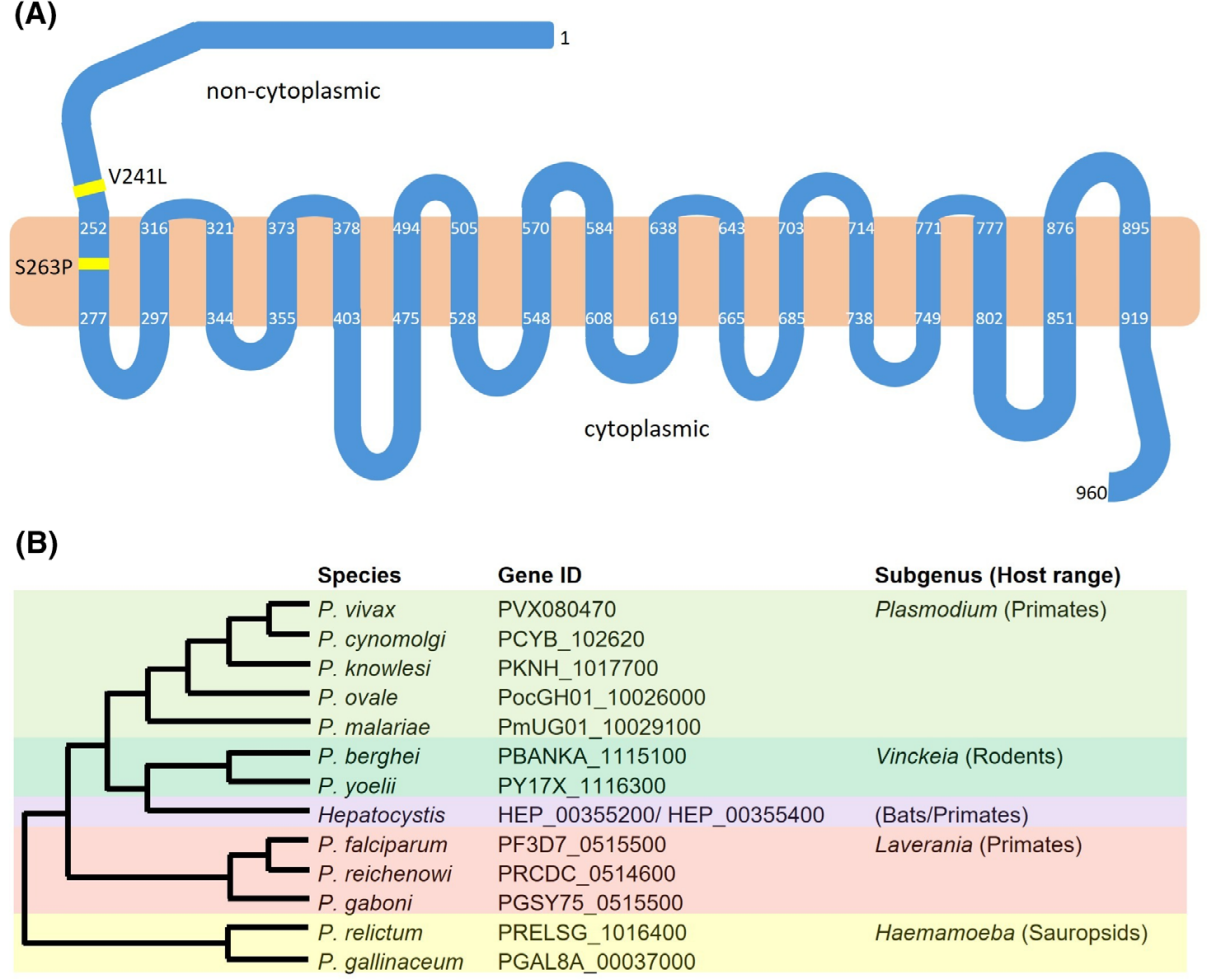
Structural prediction and orthology of *PfGEP1*. (A) *Pf*GEP1 encodes for 960 amino acids, including 17 predicted transmembrane (TM) spans. The residues defining the TMs are indicated. The amino‐terminus of approximately 250 amino acid residues and the shorter carboxyterminus are predicted to be noncytoplasmic and cytoplasmic, respectively. Two single‐nucleotide polymorphisms (SNPs), V241L and S263P, have been identified (yellow lines). (B) Representation of gametogenesis essential protein 1 (*GEP1*) orthologs across the genus *Plasmodium*. Shown are *Plasmodium* species, gene identifier (accession numbers), subgenus, and host taxa. The Mutliple Sequence Alignment Tool was used to generate the tree.

*PfGEP1* encodes a protein of 960 amino acids with 17 predicted transmembrane domains and approximately 250 amino acids reaching into a noncytoplasmic compartment at the N terminus (Fig. 1A). Its classification as a transporter was inferred by weak similarity to a family of Na^+^‐neurotransmitter symporters that transport a wide array of substrates, ranging from neurotransmitters to amino acids [16]. Accordingly, it was originally labeled neurotransmitter: Na^+^ symporter 2 (NSS2) [17]. Sequence alignment of *Pf*GEP1 with its orthologs in the *Plasmodium* genus revealed conservation within the *Laverania* subgenus and 80%–90% similarity to genes found in the *Plasmodium*, *Vinckeia*, *Hepatocystis*, and *Haemamoeba* subgenera (Fig. 1B). In *Hepatocystis*, two orthologs were identified on two separate chromosomes corresponding to half the *Pf*GEP1 protein each. These most likely represent sequence mis‐assembly in the draft *Hepatocystis* genome [18]. Outside of the genus *Plasmodium*, no orthologs of GEP1 can be identified based on sequence similarity. However, an *in silico* structure search (foldseek) predicts structural overlap between the *Pf*GEP1 protein and candidate transporters in a range of eukaryotic and bacteria species (Fig. S1), exemplified by the human gamma‐aminobutyric acid (GABA) transport protein SLC6A1 (Video S1). This structural similarity appears restricted to amino acid residues 300–900 of *Pf*GEP1, whereas the amino‐terminus is a unique hallmark of *Plasmodium* GEP1 proteins [19].

Two nonsynonymous single‐nucleotide polymorphisms (SNPs) in the *PfGEP1* gene were reported thus far [20, 21]. The V241L polymorphism was identified in a study searching for markers of chloroquine/quinine resistance, but no association was observed between this allele and altered drug efficacy [20]. The V241L SNP, as well as a second SNP, S263P, was later reported to be associated with reduced *in vitro* susceptibility to artemether in samples collected in Nigeria [21], but a functional link between these SNPs and drug resistance remains to be determined. AlphaFold structure prediction [22] places both variants outside a proposed XA‐binding pocket [23] (Fig. S2).

Here, we report a complete lack of gametogenesis in parasites deficient for *PfGEP1* in the human pathogen *P. falciparum*. We found this defect to appear independently of XA and to not be reversible by addition of the phosphodiesterase inhibitor Zaprinast, lending further support for the notion that GEP1 is necessary for basal activity of GCα rather than being a receptor for the external stimulus. This observed arrest in gamete activation in a human malaria parasite qualifies *Pf*GEP1 as a candidate transmission‐blocking target. We also assessed the prevalence of the known SNPs in the *PfGEP1* gene in a sample collection obtained from travelers returning from African countries with malaria.

## Methods

### Ethical approval

Blood samples were collected from returning travelers with *P. falciparum* malaria treated at Charité Universitätsmedizin Berlin between January 2015 and December 2022 within the framework of the Study on Determinants of Malaria Semi‐Immunity and Tolerance (DEMIT). The study methodologies conformed to the standard set by the Declaration of Helsinki. The study was approved by the ethics committee of Charité Universitätsmedizin Berlin (EA4/092/21). The study was undertaken with the understanding and written consent of each individual. Sampling was performed at Charité Universitätsmedizin Berlin. For parasite cultures, O^+^ erythrocytes were purchased as concentrate from the German Red Cross, and human serum (blood groups A^+^/B^+^/AB^+^) was purchased from Haema AG (Berlin, Germany).

### Parasite maintenance

*P. falciparum* parasites were maintained in RPMI1640 media (PAN Biotech, Aidenbach, Germany; containing 25 mm HEPES, and 2.0 g·L^−1^ NaHCO_3_) supplemented with 480 μm hypoxanthine and 20 μg·mL^−1^ gentamicin. Parasites were cultured in medium containing 10% w/v heat inactivated human serum (blood groups A^+^/B^+^/AB^+^, obtained from Haema AG) and in O^+^ erythrocytes (German Red Cross) at a 4% hematocrit at 37 °C and slight agitation to prevent settling of the cells. Cultures were gassed with a premade gas mixture of 5.0% CO_2_ and 3.0% O_2_ in N_2_ (Westfalen AG, Muenster, Germany). Parasite growth was monitored by microscopic examination of Giemsa‐stained blood smears. Parasites were regularly treated with sorbitol to maintain synchronicity [24].

### RNA extraction and qRT‐PCR

Parasites were freed from surrounding erythrocytes through saponin lysis [25]. RNA was extracted according to the manufacturer's protocol (Macherey Nagel, Dueren, Germany). RNA concentration was measured on a Nanodrop ND 1000 (peQLab, Erlangen, Germany), and RNA was immediately used for cDNA synthesis using the Superscript IV Reverse Transcription Kit (Thermo Fisher) with or without (control) addition of reverse transcriptase. cDNA samples were mixed with the Power Sybr Green Master Mix (Thermo Fisher, Waltham, MA, USA) according to the manufacturer's protocol; approximately 2 ng cDNA was added for each qPCR reaction. Primers were added at a final concentration of 0.5 μm. The qPCR reaction was performed using a Quantstudio 1 (Thermo Fisher) with the protocol set as follows: 95 °C for 2 min, then 40 cycles of 95° for 15 s, 56 °C for 30 s, and 60 °C for 30 s. This procedure was followed by a preset melting curve (60 °C for 1 min ramping up to 95 °C at 0.15 °C·s^−1^). The resulting *C*_t_ values were used to calculate relative expression based on the ΔΔ*C*_t_ method [26] against seryl tRNA synthetase (*hk1*), fructose‐bisphosphate aldolase (*hk2*), and *HSP70* (*hk3*).

### Generation of CRISPR‐Cas9 gene disruption plasmids

gRNAs were designed using an online tool (Benchling.com) with the following settings: single guide, 20‐bp long, map against 3D7 genome, PAM: NGG (SpCas9, 3′side). Two gRNAs with an on‐target score > 50 and an off‐target score > 90 were selected (Table S1). Primers were designed to amplify two homology regions (HR1/2) of 300–500‐bp length around the two gRNA binding sites (Table S2). Homology regions were amplified from 20 ng NF54 genomic DNA per reaction using DreamTaq Polymerase (Thermo Fisher). Homology regions were inserted into the pDC2‐hdhfr‐Cas9 plasmid backbone [27] through Gibson Assembly [28] to surround the *hDHFR* cassette at the *PspOM*I, *EcoR*I, and *Aat*II restriction sites. Successful integration of homology regions was confirmed through analytical digests using diagnostic combinations of restriction enzymes. gRNA oligonucleotides were annealed and inserted at the *Bbs*I restriction site into the pDC2‐Cas9‐hdhfr backbone containing the homology regions. Successful insertion of the gRNAs was confirmed using Sanger Sequencing (LGC Genomics, Berlin, Germany).

### Transfections

Ring stage parasites were transfected with 100 μg plasmid resuspended in 15 μL TE‐buffer [29]. At least 8 h after transfection, but before parasites completed their current replication cycle, the medium was removed and replaced with medium containing 4 nm WR99210 (Jacobus Pharmaceutical, Plainsboro, NJ, USA). Medium was changed daily for 10–14 days and then three times a week, and fresh erythrocytes were added weekly. Once parasites emerged, they were analyzed by diagnostic PCR. Recombinant parasite populations with the desired gene deletion were purified by clonal dilution and used for assays.

### Growth assay

Asexual blood stage cultures were synchronized twice with sorbitol at six ‐hour intervals [24]. The next day, the parasitemia of late‐stage parasites was calculated by microscopic examination of Giemsa‐stained blood films. From each culture, three technical replicates were set up at 0.1% parasitemia. Two days later, parasitemia was assessed by counting > 2000 erythrocytes per culture. Parasitemia of each culture was normalized against the mean parasitemia of the WT reference line NF54 for each replicate.

### Gametocyte culture

Synchronized cultures [24] were set up at 2% late‐stage parasitemia at 3% hematocrit (Day −3). The day after, half the medium was replaced by new medium (Day −2). Parasites were allowed to reach the mature schizont stage and were split to 2% just before merozoite egress (Day −1). When committed ring stages were present at the onset of the next cycle, the medium was changed to medium containing 50 mm GlcNAc (Day 0). On Day 1 of gametocyte development, cultures were treated with sorbitol. Medium was changed daily with GlcNAc medium being used for 6–8 days until no visible asexual parasites remained in the culture. Onward, GlcNAc‐free medium was used and media changed daily for at least three more days before gamete egress assays were performed.

### Exflagellation assay

Mature (Day 10 onward) gametocytes were briefly centrifuged (800 ***g***, 1 min), and 4 μL of infected erythrocytes were resuspended in 16 μL fresh medium containing either 100 μm xanthurenic acid or a 1 : 1000 dilution of dimethyl sulfoxide as control. This was done while maintaining the parasites at 37 °C. Parasites were then incubated for 12 min at room temperature before exflagellation centers were counted microscopically. Exflagellation centers were quantified over 25 fields of view (400× magnification) of equally distributed cells.

### Macrogamete assays

Activation of female gametocytes was induced similar to the male cells, but incubated for 2 h at room temperature, after which cells were briefly centrifuged (800 ***g*** for 1 min.) and stained with mouse anti‐*Pf*s25 (1 : 500 in PBS) and Hoechst (1 : 1000 in PBS) for 30 min. at 4 °C. Cells were washed once with PBS and then imaged using a Zeiss AxioImager fluorescence microscope. Rounded, DAPI‐ and Pfs25‐positive cells were counted as macrogametes in 25 fields of view (400× magnification) of equally distributed cells.

### Staining of activated gametocytes

Activated mature gametocytes were carefully layered on a coverslip, air‐dried, and fixed in MeOH at −80 C for 10 min. Cells were permeabilized with 0.05% saponin in 1% BSA/PBS for 30 min at room temperature and washed three times with 0.01% saponin in 1% BSA/PBS (blocking solution) before primary antibody (anti‐tubulin, mouse DM1α; Sigma Aldrich, T6199) was added (1 : 500) for 2 h at room temperature. Cells were washed again three times using blocking solution, and the secondary antibody [Alexa Fluor goat α‐mouse 488 (LI‐COR 926–68 070); 1 : 1000 in blocking solution] was added for 45 min at room temperature. After washing again three times in blocking solution, conjugated mouse α*Pf*s25 antibody was added (1 : 200 in blocking solution) for 30 min at room temperature. Cells were washed again three times and mounted using DAPI Fluoromount (Southern Biotech, Birmingham, AL, USA). From each sample, > 100 gametocytes were analyzed for quantification.

### *PfGEP1* amplicon sequencing

gDNA was isolated from 200 μL blood using the QIAamp® DNA Blood Mini Kit (Qiagen, Venlo, Netherlands) according to the manufacturer's protocol. The region containing the described SNPs was amplified using primers G47ExF and G47ExR [21] (Table S2). All positive samples were Sanger‐sequenced (LGC Genomics, Berlin, Germany) using the amplification primers.

## Results

### Expression profiling of *PfGEP1* in cultured erythrocytes

**Fig. 2:**
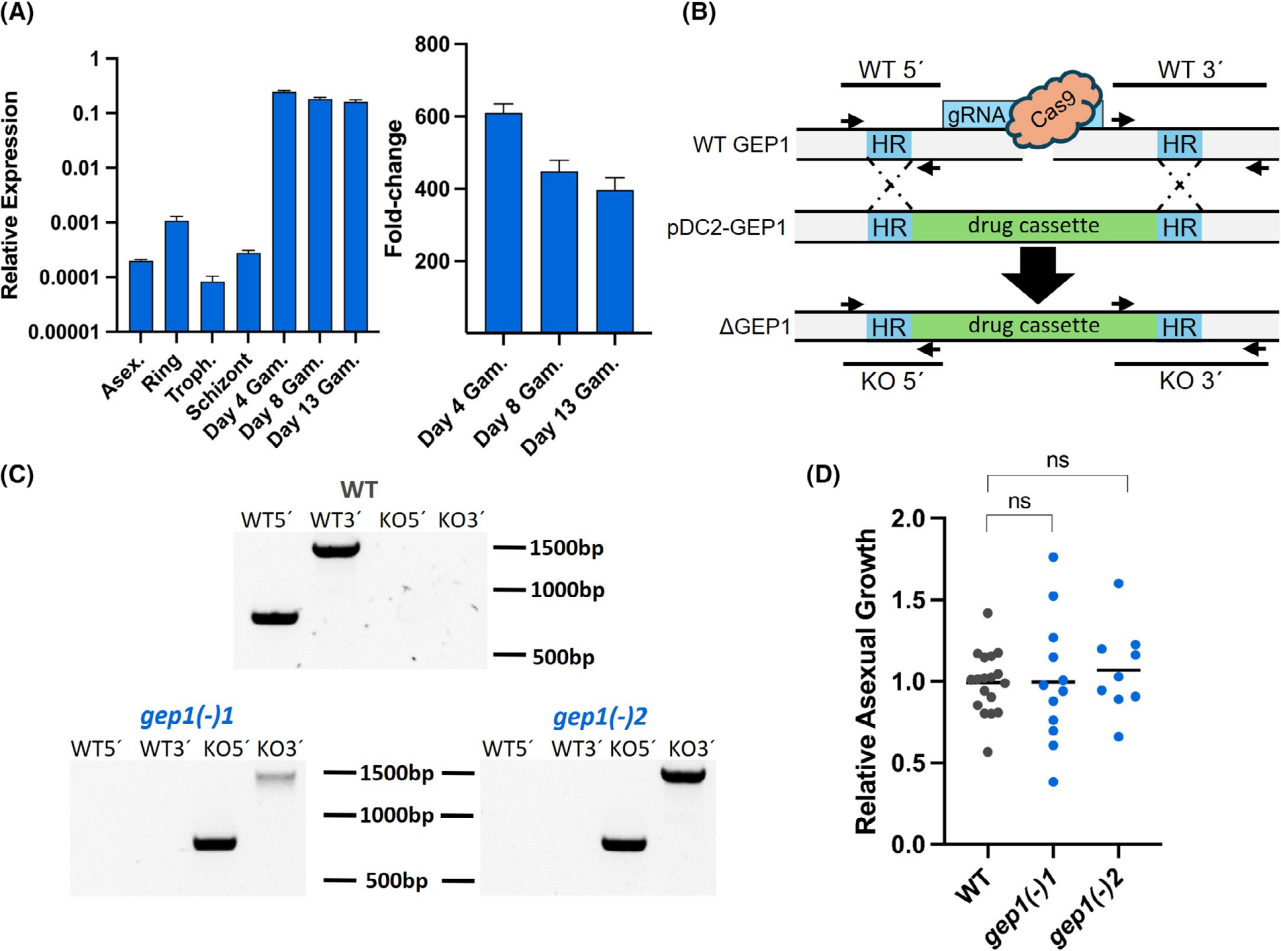
Expression profiling and targeted deletion of *PfGEP1*. (A) RT‐qPCR analysis of *PfGEP1* expression during asexual and sexual blood infection. Shown are relative expression levels (ΔΔ*C*_t_ values) (± S.D.) compared with three housekeeping genes (seryl tRNA synthetase, fructose‐bisphosphate aldolase, and *HSP70*) (left). Mixed asexual stages, synchronized ring stages, synchronized trophozoites, synchronized schizonts, and gametocytes were harvested 4, 8, and 13 days after induction and the fold change of *PfGEP1* expression in gametocytes in comparison with mixed asexual stages determined (right). Data are from one sample set done in three technical replicates. (B) Disruption strategy to generate *gep1(−)* parasite lines using a Crispr‐Cas9‐based approach. Shown are the wild‐type (WT) genomic locus, the targeting plasmid (pDC2‐GEP1), and the predicted recombinant locus after homologous recombination (ΔGEP1). Homology regions (HR, blue), the drug resistance cassette (green) for positive selection, diagnostic primers (arrows), and PCR products (lines) are indicated. (C) Diagnostic PCR to verify successful *PfGEP1* disruption in two separate cell lines. Primer combinations and PCR products as indicated in B. (D) Asexual growth of *gep1(−)* cell lines relative to NF54 WT parasites. Growth was monitored over 48 h and shown normalized to NF54 growth rates. n.s., nonsignificant (*P* > 0.05; *t*‐test, three biological replicates with three technical replicates each).

We initiated our study by assessing the steady‐state transcript profiles of *PfGEP1* by RT‐qPCR of selected asexual and sexual blood stages relative to three housekeeping genes, namely fructose‐bisphosphate aldolase (PF3D7_1444800), seryl tRNA synthetase (PF3D7_0717700), and HSP70 (PF3D7_0818900). This analysis revealed low expression during asexual blood stages and a marked increase throughout all gametocyte stages (Fig. 2A). Interestingly, the highest expression was found in early gametocytes (Day 4), and expression decreased towards mature gametocytes. Together, the expression data indicate that *PfGEP1* likely plays a minor role during asexual blood infection *in vitro* and has an important function during gametogenesis, which is not necessarily limited to the final stages of gamete activation.

### *PfGEP1* is dispensable in asexual blood stages

Next, two CRISPR/Cas9‐based plasmids were generated to disrupt the *PfGEP1* gene in cultured *P. falciparum* parasites (Fig. 2B). Parasites were visible 26 and 49 days after transfection with the respective plasmids. Successful disruption of *PfGEP1* was confirmed by diagnostic PCR. The parasite populations originated from two independent guide RNAs in the CRISPR/Cas9 targeting plasmids and appeared isogenic. We selected two respective clonal lines, termed *gep1(−)‐1* and *gep1(−)‐2*, which exhibited the desired genotype, validating successful *PfGEP1* deletion (Fig. 2C).

Clonal parasite lines were examined for asexual blood replication at 48 h (Fig. 2D). In these growth assays, no differences of *gep1(−)‐1* or *gep1(−)‐2* growth were observed compared with NF54 WT parasites.

### *PfGEP1* does neither affect gametocyte commitment nor maturation

**Fig. 3:**
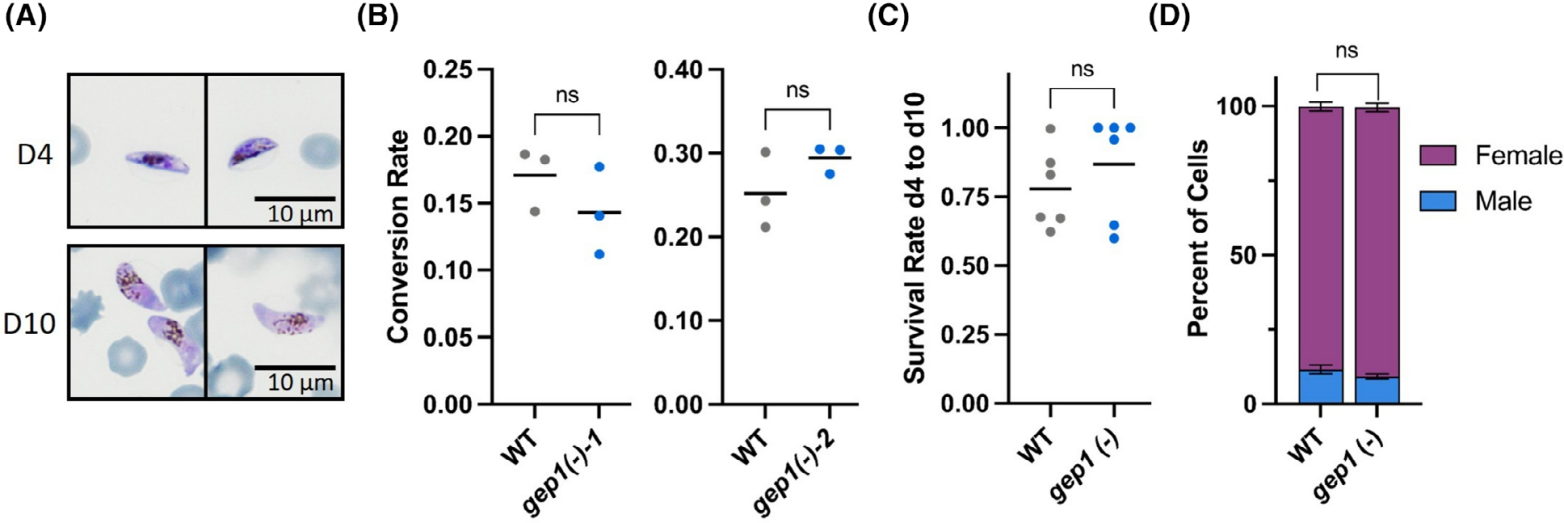
Disruption of *PfGEP1* does not affect gametocyte development. (A) Developing (top) and mature (bottom) gametocytes produced by *gep1(−)* parasite lines. (B) Comparison of gametocyte commitment between *gep1(−)* and wild‐type (WT) parasites. WT and *gep1(−)*‐1 and *gep1(−)*‐2 parasites, respectively, displayed similar gametocyte conversion rates. Parasitemias quantified in > 2000 erythrocytes, each graph represents one biological replicate with three technical replicates. (C) Survival rate of Day 4 gametocytes to reach full maturity. *Gep1(−)* and WT parasites displayed similar rates of full gametocyte maturation. Parasitemias quantified in > 2000 erythrocytes, shown are two biological replicates with three technical replicates each. Rates above 1 were set to 100% survival. (D) Sex ratio of female and male gametocytes (± S.D.). No differences in the proportion of male‐to‐female ratios were detected between mature *gep1(−)* and WT gametocytes. > 100 parasites assessed per culture, three biological replicates. n.s., *P* > 0.05 (*t*‐test).

To characterize sexual differentiation of *gep1(−)* parasites, gametocyte commitment was induced and ring stage parasites (sexually and asexually committed) were counted. Four days later, when asexual parasites had been removed, early gametocyte parasitemia was assessed and the commitment rate calculated (Fig. 3A,B). While commitment differed between repeats, no significant difference in conversion rate between WT and *gep1(−)* parasites was observed (Fig. 3B). We next assessed the rate of Day 4 gametocytes that reached maturity on Day 10 (Fig. 3A,C). The majority of gametocytes matured, and there was no difference between WT and *gep1(−)* parasites. We also enumerated the ratio of male and female gametocytes (Fig. 3D). Again, there was no apparent difference between WT and *gep1(−)* parasites. Together, we show that absence of *Pf*GEP1 does not interfere with sexual differentiation of cultured *P. falciparum* parasites.

### *PfGEP1* is essential for male gametogenesis

**Fig. 4:**
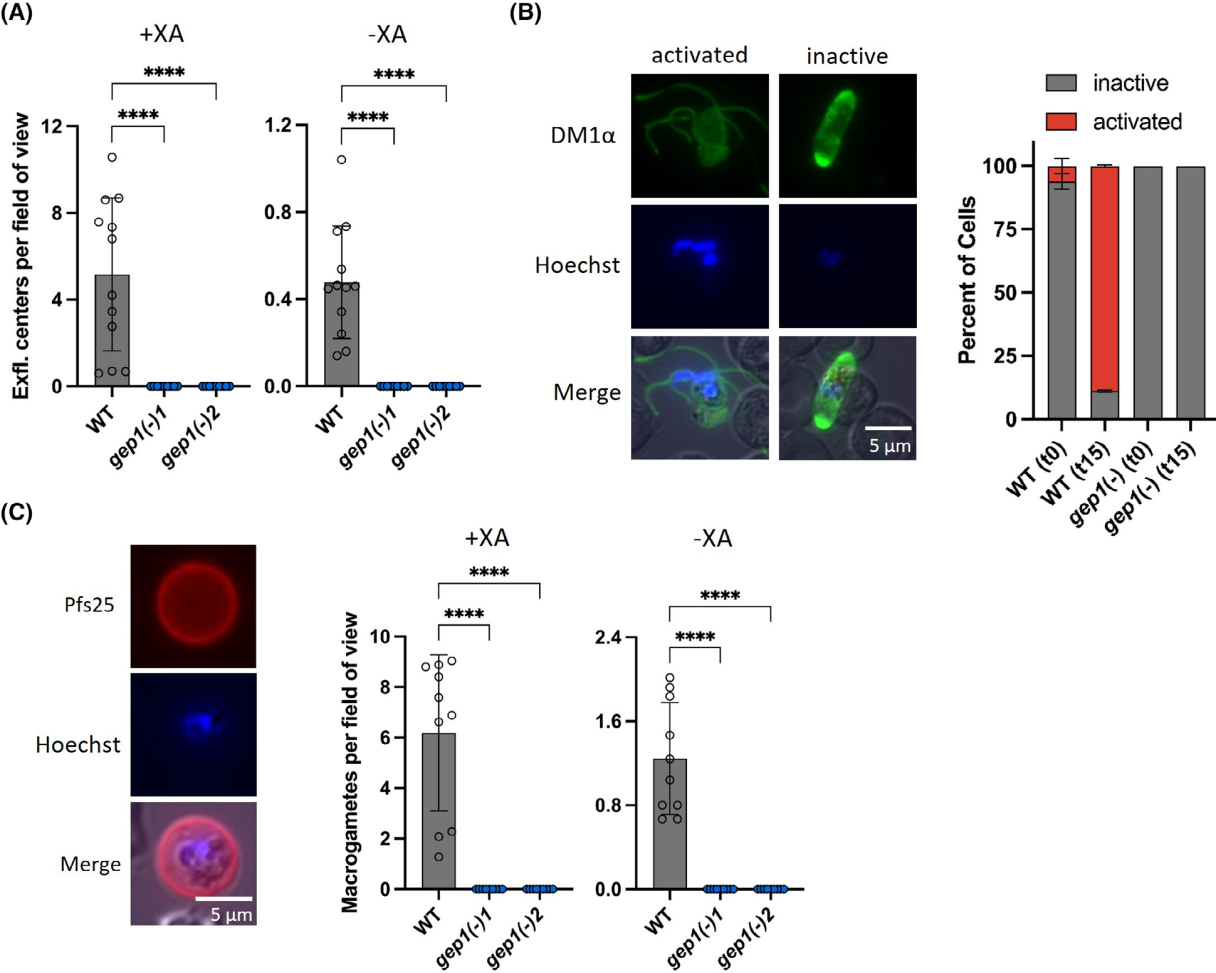
*gep1(−)* parasites display a complete arrest prior to becoming male or female gametes. (A) Enumeration of exflagellation centers (± S.D.) per field of view (400× magnification) in the presence (left) or absence (right) of 100 μm xanthurenic acid (XA). Twenty‐five fields of view assessed per replicate, shown are four biological replicates with three technical replicates each. (B) Mature gametocytes were fixed before or after 15 min with 100 μm XA activation, stained with a DM1α antibody (green) and Hoechst (blue), and the ratio (± S.D.) of activated (red) and nonactivated gametocytes (gray) were quantified (right) (> 100 parasites quantified, three technical replicates). Shown are representative images of DM1α antibody (top), Hoechst (center), and merge (bottom) of an activated (left) and a nonactivated (right) wild‐type (WT) gametocyte. (C) Mature gametocytes were activated for 2 h and macrogametes stained with a *Pf*s25 antibody (red) and Hoechst (blue). Shown (left) are representative images of *Pf*s25 antibody (top), Hoechst (center), and merge (bottom) of a WT macrogamete. Enumeration of macrogametes (± S.D.) per field of view (400× magnification) in the presence (center) or absence (right) of 100 μm XA. Twenty‐five fields of view assessed per replicate; shown are three biological replicates with three technical replicates each and an additional biological replicate with only one technical replicate. ****, *P* < 0.001 (*t*‐test).

To mimic host switch during the mosquito blood meal, we induced exflagellation in mature gametocytes with or without addition of 100 μm xanthurenic acid (XA) (Fig. 4). NF54 WT parasites produced exflagellation centers under both conditions. Without XA, WT parasites displayed substantial residual activation, which could be increased 10‐fold by XA addition (Fig. 4A). Strikingly, *gep1(−)* parasites displayed a complete absence of exflagellation, and this defect was independent of XA addition.

The absence of exflagellation events in *gep1(−)* parasites was confirmed through immunofluorescence assays using a tubulin antibody (DM1α) on cells fixed either immediately or after incubation with XA for 15 min (Fig. 4B). As expected, exflagellation centers in WT parasites were readily detected, and quantification revealed approximately 90% activation in WT parasites. In nonactivated samples, a background activity of approximately 5% was quantified in WT parasites. Notably, the gametocytes visible in the *gep1(−)* samples were neither rounded up nor did they show any other signs of activation. In conclusion, male gamete exflagellation, including XA‐independent activation, was completely abolished in the absence of *PfGEP1*.

### *PfGEP1* is essential for female gametogenesis

Activation of female gametocytes was assessed during a prolonged 2‐h activation either with or without XA. To this end, cells were stained with an antibody against the zygote surface marker *Pf*s25 and DAPI (Fig. 4C). Quantification using fluorescence microscopy revealed an average of 6.2 activated female WT gametes per field of view in the presence of XA (Fig. 4C). In the absence of XA, activation of female WT gametes was reduced to 1.2 per field of view (Fig. 4C). In marked contrast and in good agreement with the complete defect in male exflagellation, activated female gametes were completely absent in the *gep1(−)* samples (Fig. 4C).

### The *gep1(−)* defects cannot be rescued by the phosphodiesterase inhibitor Zaprinast

**Fig. 5:**
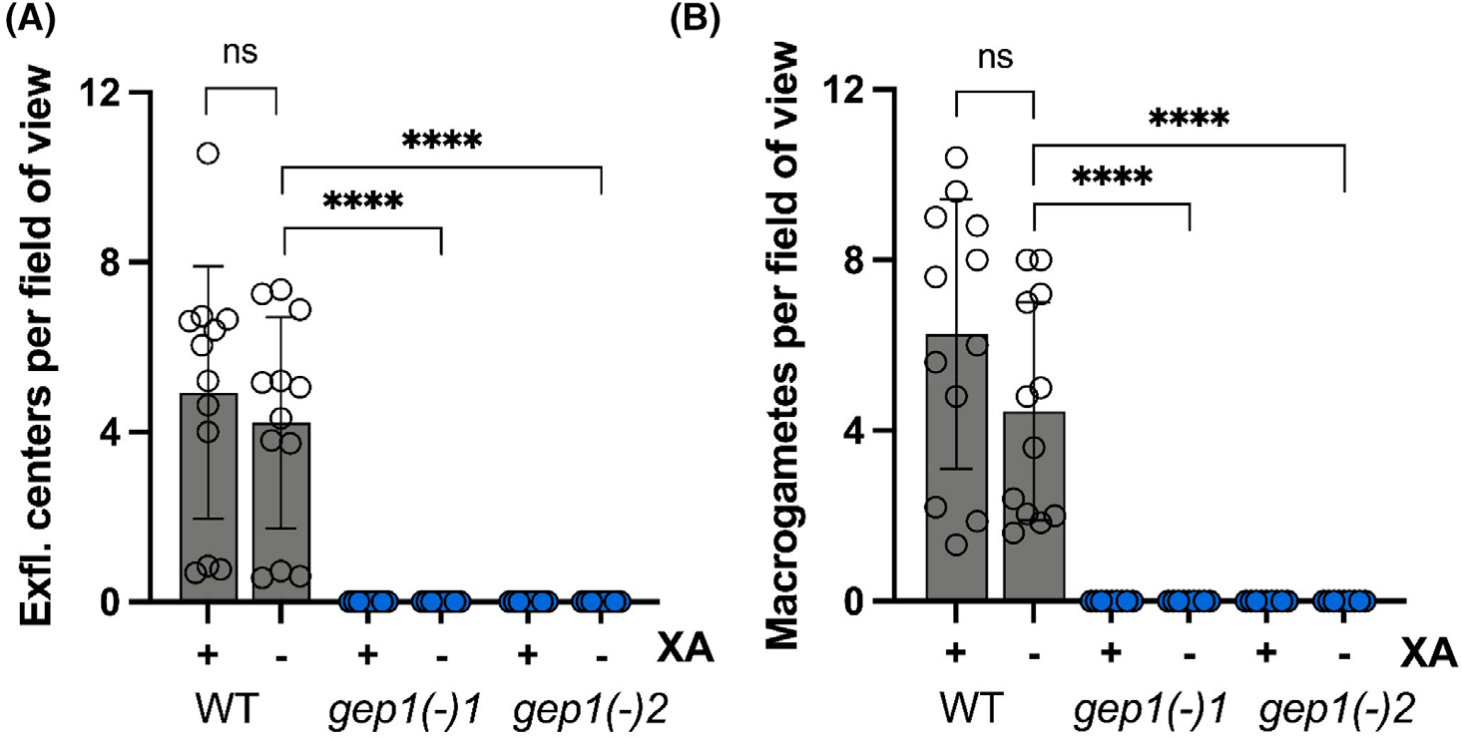
Phosphodiesterase inhibitor Zaprinast does not rescue defects of gamete activation in *gep1(−)* parasites. (A) Quantification of exflagellation centers (± S.D.) in the presence of 400 μm Zaprinast, with or without addition of 100 μm XA. (B) Quantification of macrogametes (± S.D.) in the presence of 400 μm Zaprinast, with or without addition of 100 μm XA. A/B: 25 fields of view assessed per replicate, shown are four biological replicates with three technical replicates each. ****, *P* < 0.001 (*t*‐test). XA, xanthurenic acid.

We next tested whether the gametogenesis defect of *gep1(−)* parasites can be, at least partially, overcome by addition of a cyclic guanosine monophosphate (cGMP)‐specific phosphodiesterase (PDE) inhibitor. Accordingly, mature gametocytes were activated in the presence of 400 μm Zaprinast, and assays were again performed with or without addition of 100 μm XA (Fig. 5). In good agreement with an increase in cGMP steady‐state levels, Zaprinast did not modify the number of exflagellation events of WT parasites in the presence of XA, but elevated exflagellation to approximately 4.2 per field of view without XA (Fig. 5A). A similar effect was observed when assessing macrogametes, where the addition of Zaprinast increased the number of gametes produced to 4.5 gametes per field of view without XA (Fig. 5B). In marked contrast, *gep1(−)* parasites remained unable to produce either male or female gametes under any of the tested conditions indicating that cGMP levels in these cells cannot be elevated by a cGMP‐specific PDE inhibitor and bypass the critical role of *Pf*GEP1 in gamete activation.

### Genetic diversity of *PfGEP1* in field samples

We finally employed an amplicon sequencing protocol and assessed the prevalence of the V241L and S263P polymorphisms in a collection of 52 clinical samples opportunistically collected from travelers returning from 15 different, mostly Western African, malaria‐endemic countries. GEP1 amplicons were successfully generated with a GEP1SNP primer pair for 49 samples (Table 1). Sanger sequencing revealed the V241L allele in 6 samples (12%), while the S263P allele was identified in 10 samples (20%). Notably, three samples (6%) exhibited both SNPs. In our limited dataset, we observed the two SNPs across Sub‐Saharan Africa, including Kenya and Rwanda as well Côte d'Ivoire and Gambia. We suggest that *GEP1* SNPs are widespread and moderately frequent.

**Table 1:** Prevalence of *GEP1* SNPs in samples collected from returning travelers.

Country of origin	Samples	V241L^+^	S263P^+^	V241L^+^/S263P^+^
Nigeria	14	0	2	1
Cameroon	10	1	1	0
Ghana	4	0	1	0
Togo	4	1	0	0
Benin	3	0	0	0
Burkina Faso	2	0	0	1
CAR	2	0	0	0
Guinea	2	0	1	0
Côte d'Ivoire	2	0	0	1
Sierra Leone	1	0	0	0
South Africa	1	0	0	0
Gambia	1	0	1	0
Rwanda	1	0	1	0
Congo	1	0	0	0
Kenya	1	1	0	0
Total	49	3	7	3

## Discussion

Blocking transmission of *Plasmodium* from the human host to the *Anopheles* vector is one of the main goals of malaria control and remains a research priority. Gametocytes spend most of their maturation time in the bone marrow and frequently escape detection [30]. The protracted maturation of *P. falciparum* gametocytes over the course of 10 days offers a broad window of opportunity for drug interference. Here, we were able to show that loss of function of *PfGEP1* completely blocks gametogenesis of both male and female gametocytes, fully supporting data from the *P. yoelii* murine malaria model [14]. Based on the colocalization with GCα, *Py*GEP1 was suggested to interact with GCα and act as a regulator of GCα activity. Our data are in line with the notion that *Pf*GEP1 is necessary for the basal activity of GCα, since the *gep1(−)* defect could not be bypassed with Zaprinast. The basal activity of GCα should be sufficient to elevate cGMP levels above the threshold when PDEs are inhibited, even in the absence of external cues leading to GCα activation [15]. We note that peak expression of *GEP1* mRNA is early in this process at Day 4, about a week before gametogenesis is complete. Future studies are warranted to determine the tempo‐spatial expression dynamics of GEP1 and potential functions prior to the terminal steps.

Recently, two additional proteins have been implicated in GCα activity, termed signaling linking factor (SLF) and unique GC organizer (UGO) [31]. Similar to *gep1(−)* lines, parasites deficient for *SLF* or *UGO* showed impaired gametogenesis. Interestingly, this defect could be bypassed by Zaprinast in *UGO*‐deficient parasites, but not in those lacking *SLF* [31]. Together, it appears that GEP1 and SLF are required for efficient GCα activity in mature gametocytes, either by direct binding or upstream of GCα activation. In contrast, UGO appears to elevate GCα activity and trigger gametogenesis in response to external stimuli in the mosquito midgut, making UGO currently the best candidate for the long‐sought XA receptor.

Experimental genetics in *P. yoelii* initially assigned this role to *GEP1* [14]. This hypothesis was further supported by structural analysis done on the interactions between GEP1, XA, and GCα, which postulated a candidate XA‐binding pocket in the GEP1 protein [23]. In WT parasites, we observed activation of both male and female gametocytes independently of XA, most likely caused by the drop in temperature and an elevated pH upon removal from cultures resulting in a reduced PDE activity [30]. Strikingly, this basal level of XA‐independent activation was also entirely absent in *gep1(−)* parasites, indicating that direct XA binding, if any, is unlikely the exclusive function of GEP1.

In this study, we analyzed the prevalence of two GEP1 nonsynonymous SNPs, V241L, and S263P in parasite isolates from patients with malaria. The SNPs were originally described from Nigeria [21], where the prevalence reached 25%. We found both SNPs in samples from various African countries, and the SNPs were moderately abundant, including several samples that were both V241L^+^ and S263P^+^. One study suggested that both SNPs might contribute to reduced artemether susceptibility [21]. Given the distinct defect of *gep1(−)* parasites in the final step of *P. falciparum* gamete maturation combined with the remarkably low expression level observed in asexual parasites shown in this study and by others [32], we consider it highly unlikely that *GEP1* mutations confer drug resistance during asexual development. We instead propose that allele diversity in *GEP1* might influence onward transmission to the *Anopheles* vector and future malaria episodes.

In conclusion, our experimental genetics analysis in *P. falciparum* qualifies *Pf*GEP1 as a transmission‐blocking target. To further examine this potential for targeted drug development, biochemical studies are needed to assign a catalytic and/or regulatory activity to *Pf*GEP1. Since *Pf*GEP1 is a membrane‐spanning protein, the precise localization and cellular compartment will be critical determinants for a better molecular understanding of GPE1 functions. Its annotation as a transporter [17] warrants strategies to identify the GEP1 cargo. Whether GEP1 can be targeted by a drug also depends on the hitherto unknown functional role(s) of the amino‐terminus, which contains two closely adjacent nonsynonymous SNPs, widespread in endemic parasite populations across Sub‐Saharan Africa.

## Author contributions

FH, AGM, and KM designed and conceptualized the experiments. FH, MSC, and LSL performed the experiments. FK generated the blood sample collection; FH, FK, AGM, and KM analyzed and interpreted the data. FH and KM wrote the manuscript. All authors contributed to the article and approved the submitted version.

## Supporting information


**Fig. S1.** Foldseek structure search with *Pf*GEP1.
**Fig. S2.** alphafold prediction of the V241L and S263P variants.
**Table S1.** gRNAs.
**Table S2.** Primers used in this study.

**Video S1.** Animation of structural alignment of PfGEP1 and human GABA transport protein SLC6A1.

## Data Availability

Additional data are available in [Link], [Link].
