# Supplementary material for: Plasmodium falciparum gametogenesis essential protein 1 (GEP1) is a transmission‐blocking target
Source: FEBS Lett. 2025 Oct 13;600(2):239–50. doi: 10.1002/1873-3468.70184 (PMC12834003; doi:10.1002/1873-3468.70184)
Supplement: Supplementary file 1 — Fig. S1. Foldseek structure search with PfGEP1. Fig. S2. alphafold prediction of the V241L and S263P variants. Table S1. gRNAs. Table S2. Primers used in this study. [file FEB2-600-239-s001.pdf]

Supplemental Information for:

Plasmodium falciparum gametogenesis essential protein 1 (GEP1) is a transmission blocking target

Frederik Huppertz, Milagros Siebeck Caturelli, Lina S. Lehmann, Florian Kurth, Alexander G. Maier, Kai Matuschewski

Content:

Figures S1, S2

Video S1

Tables S1, S2

Huppertz *et al.*, Supplemental Figure S1

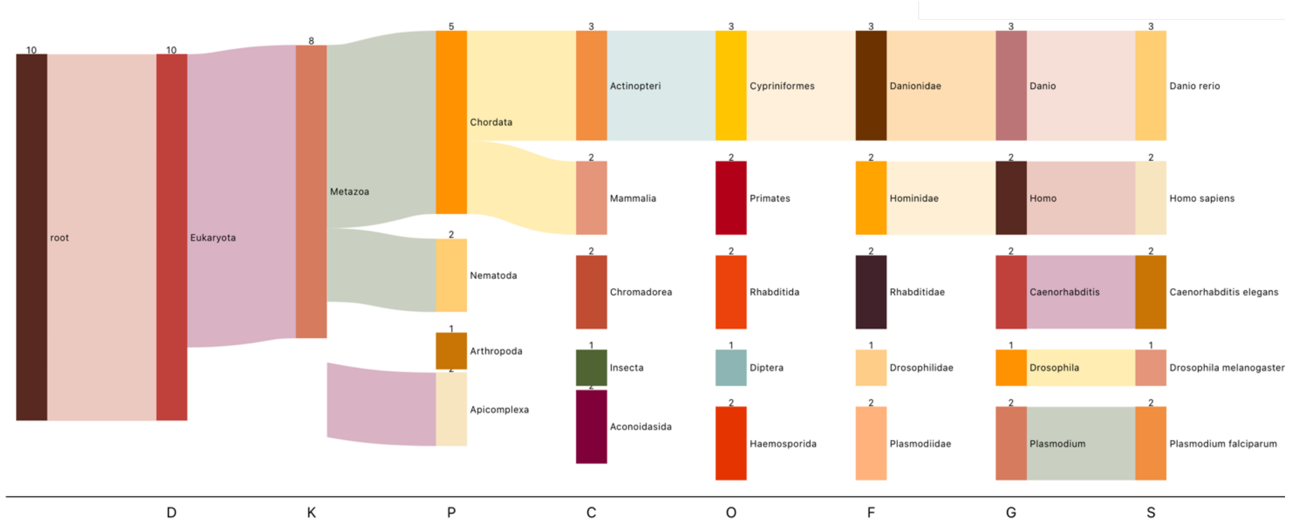

Supplemental Figure S1: Taxonomy Tree of *Pf*GEP1-related protein structures generated by FoldSeek.

Supplemental Video S1: Animation of overlay of the human GABA transport protein SLC6A1 (grey) and *Pf*GEP1 (blue) displaying similar folds despite absence of similarity in primary amino acid sequence.

Huppertz *et al.*, Supplemental Figure S2

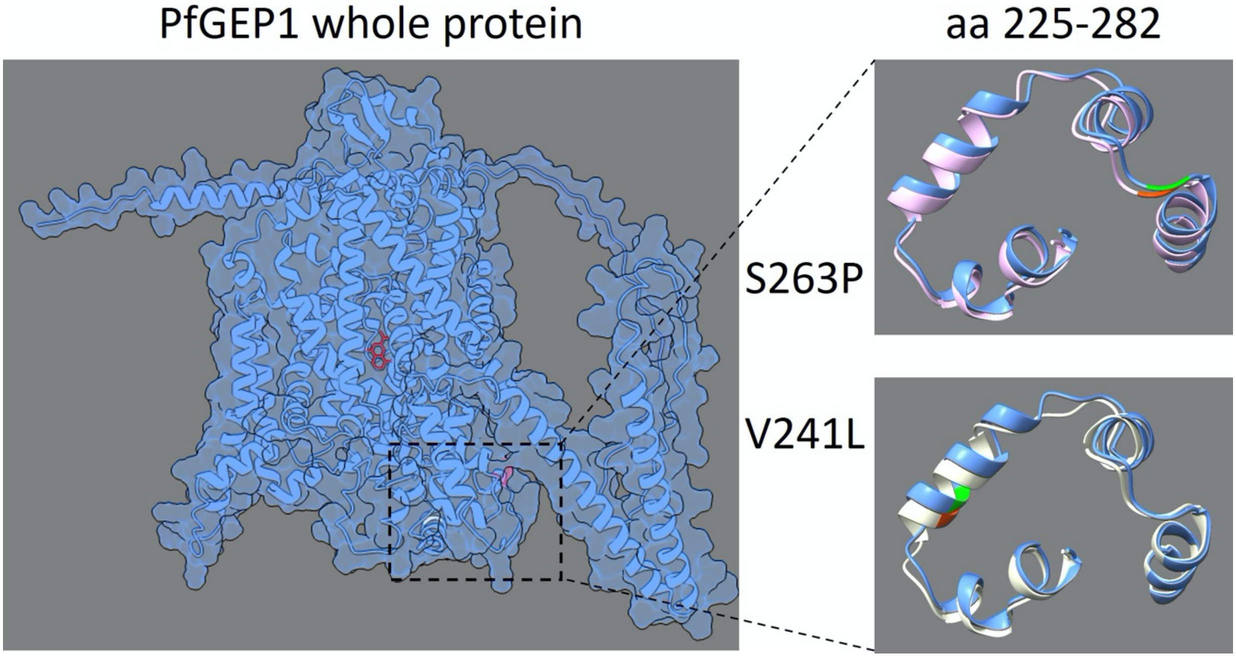

Supplemental Figure S2: AlphaFold predictions of wild-type *Pf*GEP1 in the presence of xanthurenic acid (red) (left) and amino acid residues 225-282 of the two variants (right), S263P (top) and V241L (bottom). Wild-type and variant amino acid residues are colored in green and red, respectively.

Table S1: gRNAs

Name	Plus strand	Minus strand
1070	TATTTCCATCTAATACAAAACGTC	AAACGACGTTTTGTATTAGATGGA
1128	TATTAATAATTACCATCTACCTAT	AAACATAGGTAGATGGTAATTATT

Cloning overhangs are shown in red

Table S2: Primers used in this study

Primer	Forward	Reverse
GEP1_qpcr	GGTGATGATTATTTCCCCTTTAGG	GATTATTTCTGAAGTTACAAATTTTGG
Hk1_qpcr	TGGTTATTATTTAACAGGTGCAGG	CAAGTTCGGCACATTCTTCC
Hk2_qpcr	TCAATGCTTTGGGTCCACAC	CTAAGGAGTTGGCTTCAGCTCT
Hk3_qpcr	TCTATCAAGATGCTGCTGGTGC	GCATTTCCTGGCATTCCTGC
GEP1_HR1	GAATACCAAAATTTGTAACTTCAG	ATGGGAATATGATAGCAAATG
GEP1_HR2	CTCTCTACACCTTCTATACCTTTC	TAGCATACACAATATCCAGACT
GEP1i_5´	GAATATAAATAATTATAAATGTTCAAACGTG	TCCATCTAATACAAAACGTCCGG
GEP1i_3´	CTGGTCAATAAATAATTACCATCTACC	CCTTTGGAGGTGTAGAAATATAATCC
pDC2i (3´Fw and 5´Rv)	CATTATATATAAGGACATATTTATTAAACCGC	CATAAACGAGAGTACCTCTTACAAGCG
GEP1_SNP	GTATAGATATTAAAGATGCC	CATATTTTCAAATACACTCGCCATA
